# Metagenomic Next-Generation Sequencing Successfully Detects Pulmonary Infectious Pathogens in Children With Hematologic Malignancy

**DOI:** 10.3389/fcimb.2022.899028

**Published:** 2022-06-28

**Authors:** Dao Wang, Weilin Wang, Yanjie Ding, Miaomiao Tang, Lei Zhang, Jiao Chen, Hongliang You

**Affiliations:** Department of Pediatrics, The First Affiliated Hospital of Zhengzhou University, Zhengzhou, China

**Keywords:** hematologic malignancy, children, pulmonary infection, mNGS, diagnosis

## Abstract

**Background:**

Pulmonary infection is a leading cause of mortality in pediatric patients with hematologic malignancy (HM). In clinical settings, pulmonary pathogens are frequently undetectable, and empiric therapies may be costly, ineffective and lead to poor outcomes in this vulnerable population. Metagenomic next-generation sequencing (mNGS) enhances pathogen detection, but data on its application in pediatric patients with HM and pulmonary infections are scarce.

**Methods:**

We retrospectively reviewed 55 pediatric patients with HM and pulmonary infection who were performed mNGS on bronchoalveolar lavage fluid from January 2020 to October 2021. The performances of mNGS methods and conventional microbiological methods in pathogenic diagnosis and subsequently antibiotic adjustment were investigated.

**Results:**

A definite or probable microbial etiology of pulmonary infection was established for 50 of the 55 patients (90.9%) when mNGS was combined with conventional microbiological tests. The positive rate was 87.3% (48 of 55 patients) for mNGS versus 34.5% (19 of 55 patients) with conventional microbiological methods (*P* < 0.001). Bacteria, viruses and fungi were detected in 17/55 (30.9%), 25/55 (45.5%) and 19/55 (34.5%) cases using mNGS, respectively. Furthermore, 17 patients (30.9%) were identified as pulmonary mixed infections. Among the 50 pathogen-positive cases, 38% (19/50) were not completely pathogen-covered by empirical antibiotics and all of them were accordingly made an antibiotic adjustment. In the present study, the 30-day mortality rate was 7.3%.

**Conclusion:**

mNGS is a valuable diagnostic tool to determine the etiology and appropriate treatment in pediatric patients with HM and pulmonary infection. In these vulnerable children with HM, pulmonary infections are life-threatening, so we recommend that mNGS should be considered as a front-line diagnostic test.

## Introduction

Pulmonary infection is a leading cause of mortality in pediatric patients with hematologic malignancy (HM), particularly those receiving high dose chemotherapy or hematopoietic stem cell transplantation (HSCT) (Guidelines for Preventing Infectious Complications Among Hematopoietic Cell Transplant Recipients: A Global Perspective, 2009; Morrison, 2010). Due to the significant immunosuppression mainly caused by their treatments, these patients are vulnerable to infections. Approximately 30% of patients with HM experience pulmonary infiltrates during their management, therefore associated with higher mortality up to 50% (Ewig et al., 1998). And the incidence reaches 70% in HSCT recipients (Harris et al., 2016), with a mortality of up to 50% (Wang et al., 2004). However, many possible pathogens make its pathogenic diagnosis challenging, including some common and uncommon pathogens ranging from bacteria to viruses, fungi, and parasites (Morrison, 2010; Renaud and Campbell, 2011; De La Cruz and Silveira, 2017).

Prompt and precise pathogenic diagnosis of pulmonary infections facilitates the timely application of optimal antimicrobial therapy, which may save precious time for the treatment and achieve satisfactory clinical improvement in these patients with a life-threatening disease. The current microbiological methods, mainly including microscopy, culture, serology and polymerase chain reaction-based pathogen-specific nucleic acid detection (Sugawara et al., 2013), have limitations in many aspects, such as the limited breadth of pathogens detected, long culture time, low specificity and low sensitivity (Gu et al., 2019), which hinder rapid diagnosis and precise treatment.

As the rapid development in sequencing technology and bioinformatics (Popovich and Snitkin, 2017; Thoendel et al., 2018; Rossen et al., 2018), metagenomic next-generation sequencing (mNGS) has been increasingly applied in clinical practice (Salzberg et al., 2016; Pendleton et al., 2017; Chen et al., 2020). mNGS approaches identify potential pathogens through direct DNA and RNA sequencing and database comparison of clinical samples without the need for culture and premise hypothesis. Compared to conventional pathogen detection methods, mNGS offers distinct advantages for broad-spectrum detection of common and uncommon pathogens. However, reports on the application of mNGS in pulmonary infections among pediatric patients with HM remain scarce.

We retrospectively analyzed the mNGS results from bronchoalveolar lavage fluid (BALF) obtained from 55 pediatric patients with HM and pulmonary infection to evaluate the diagnostic value of mNGS in these patients compared to conventional microbiological methods.

## Materials and Methods

We retrospectively reviewed 73 pediatric patients with HM and pulmonary infection at the pediatric hematology department, the First Affiliated Hospital of Zhengzhou University from January 2020 to October 2021. With our inclusion criteria, 55 pediatric patients were enrolled for this study. The inclusion criteria were as follows; 1): children with HM were diagnosed with pulmonary infection based on the criteria of clinical management guidelines of the World Health Organization (Organization W H., 2005); 2) all children underwent bronchoscopy to obtain BALF; 3) both mNGS and conventional microbiological methods were used to detect pathogens; 3) children under 16 years old; 4) complete clinical history. Besides, we collected the patients’ clinical characteristics, including clinical symptoms, laboratory test results, imaging examination results, diagnosis, treatment process and prognosis.

mNGS assays for the BALF samples were performed in all patients, paralleled with the conventional microbiological assays. The conventional microbiological assays were as follows: bacterial and fungal smear and culture, serum antibodies for indirect immunofluorescence assay for respiratory syncytial virus (RSV), influenza A/B virus, parainfluenza virus, adenovirus, coxsackievirus, Epstein-Barr virus (EBV), cytomegalovirus (CMV) and mycoplasma pneumonia (MP), chlamydia pneumonia were performed. Galactomannan enzyme immunoassay (GM-test) and 1, 3) β-D-glucan assay (G-test) were also performed for fungi. As for mNGS assays, DNA sequencing was performed for all 55 samples, 26 of which RNA sequencing was performed at the same time.

### Sample Preparation, DNA/RNA Extraction, Library Construction and Sequencing

6-10ml BALF samples were collected from patients according to the standard clinical procedure. DNA from BALF samples was extracted using the QIAamp^®^ UCP Pathogen Kit (Qiagen, Germany) according to the manufacturer’s recommendations. RNA from BALF samples was extracted using the TIANamp Virus RNA DP315-R Kit (Tiangen Biotech, Beijing, China) following the manufacturer’s instructions.

The extracted DNA samples were used to construct DNA libraries by using the TruePrep DNA Library Prep Kit V2 for Illumina^®^ (Vazyme, Nanjing, China). RNA libraries were prepared from the extracted RNA samples by using the VAHTS Universal RNA-seq Library Prep Kit V6 for Illumina^®^ (Vazyme, Nanjing, China). All libraries were prepared following the manufacturer’s manuals. The Agilent 2100 Bioanalyzer (Agilent Technologies, Santa Clara, USA) was used for library quality control. All libraries were pooled with other libraries by using different index sequences and sequenced on an Illumina NextSeq 550Dx platform with the single-end 75bp sequencing option. For each run, negative control (NC) samples (Nuclease-free H_2_O) were also pooled to monitor reagent and laboratory background.

### Bioinformatics Analysis

Fastq-format data were obtained for each sample by using bcl2fastq software (v2.20.0.422, parameters used:–barcode-mismatches 0 –minimum-trimmed-read-length 50). Adapt sequences and low-quality reads were filtered out using cutadapt v2.10 (-q 25,25 -m 50). The remaining high-quality reads were first mapped to the human genome (hg38, https://hgdownload.soe.ucsc.edu/downloads.html#human) using bwa-mem 2 v2.1 with default parameters, all unmapped reads (identified as microbiome-derived sequences) were then aligned to the NCBI nt database (https://ftp.ncbi.nlm.nih.gov/genomes/) by using BLAST v2.9.0+ (-task megablast -num_alignments 10 -max_hsps 1 -evalue 1e-10). Alignments were required to be full-length with an identity of at least 95%. A customized Python script was used to identify species-specific alignments. Only the alignments that fulfill the above-mentioned criteria were used for further pathogen identification. Besides, the NC samples were used to identify reagent and laboratory contaminants. The list of microbes detected in NC samples was used for background subtraction from the list of microbes obtained in patient samples. The remaining microorganisms were considered credible if the following criteria were met; 1) the microbe had at least 3 non-redundant, mapped reads per 10 million raw sequence reads, and 2) the microbe was known to be potentially pathogenic in the given clinical context of each particular patient.

### Criteria for a Positive mNGS Result

Infectious pathogens were considered positive if any of the following criteria were met: 1) the relative abundance of pathogens detected by mNGS was greater than 30% at the genus level; 2) the pathogen also detected by conventional microbiological methods and the mNGS reads number was more than 50 from a single species; 3) at least one unique read was mapped to species or genus level for Mycobacterium tuberculosis (Li et al., 2018). It is important to note that the pathogens could not be directly determined as infection, colonization, and contamination by conventional microbiological methods and mNGS results. Thus, patients’ radiology, clinical characteristics and anti-infection outcomes were taken into consideration by three experienced physicians to make the final decision after obtaining the microbiological evidence. The clinical characteristics and mNGS data of all patients were provided in **Supplemental Table 1**.

### Statistical Analysis

All data analysis was performed using SPSS software (version 25.0) with the McNemar test. The data are expressed as median (range). *P* value < 0.05 was considered statistically significant and the test was two-tailed.

## Results

### Sample and Patient Characteristics

Finally, a total of 55 pediatric patients were enrolled, 23 of whom were diagnosed with acute lymphoblastic leukemia (ALL), 9 were acute myeloid leukemia (AML), and 23 were allo-HSCT recipients. The median patient age of 39 boys and 16 girls was 6 years (range 1–16 years). BALF was collected for mNGS at a median time of 5 days (range 3-16 days) following symptom onset. Of the 55 pediatric patients, 31 had neutropenia (56.4%). All 55 patients had already received empirical antibiotic therapy before mNGS and their conditions did not improve. Of note, oxygen therapy was required in 42 of 55 pediatric patients (76.4%), nine of which mechanical ventilation was required (**Tables 1**, **2**).

**Table 1:** Clinical characteristics of pediatric patients.

Characteristics	Count	Ratio
**Sex, No. (%)**		
Male	39	70.9%
Female	16	29.1%
**Age (year)**		
Median (range)	6 (1–16)	
**Disease classification**		
ALL	23	41.8%
AML	9	16.4%
allo-HSCT^a^	23	41.8%
**Neutropenia**	31	56.4%
**Respiratory support**		
Mechanical ventilation	9	16.4%
Face mask	11	20.0%
Nasal catheter	22	40.0%
None	13	23.6%
**Chest CT scan**		
Abnormal	55	100%
**Antibiotic use before mNGS**	55	100%
**Clinical outcome**		
Relieved	51	92.7%
Deceased	4	7.3%

BALF, Bronchoalveolar lavage fluid; ALL, Acute lymphoblastic leukemia; AML, Acute myelogenous leukemia.

a allo-HSCT: hematopoietic stem cell transplantation (three for ALL, seven for AML, eleven for aplastic anemia, one for myelodysplastic syndrome and one for chronic myelocytic leukemia).

**Table 2:** Routine laboratory test results of pediatric patients.

Test	Count
White blood cell	2.75 (0.13-15.20) ×10^9^/L
Platelet	66 (3–566) ×10^9^/L
Neutrophil	1.42 (0.01-13.64) ×10^9^/L
Lymphocyte	0.58 (0.03-5.33) ×10^9^/L
C-reactive protein	50.39 (0.50-196.00) mg/L
Procalcitonin	0.31 (0.043-30.20) μg/L

### Comparison of mNGS and Conventional Microbiological Methods

In the present study, the overall yield rate was 90.9% (50 of the 55 patients) when mNGS was combined with the conventional microbiological methods. The positive rate of pathogens detected by mNGS was 87.3% (48 of 55 patients), thus by conventional microbiological assays was only 34.5% (19 of 55 patients) (*P*<0.001).

**Figure 1:**
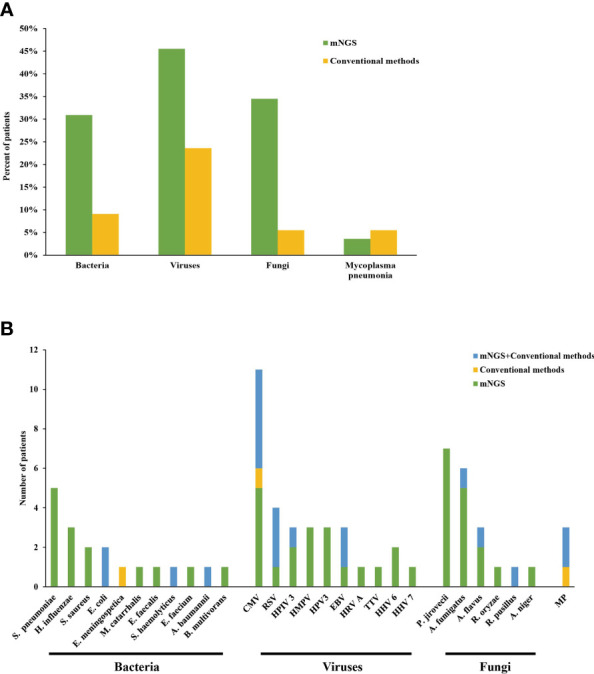
**(A, B)** Distribution of pathogens detected by mNGS and conventional methods. S. pneumoniae, Streptococcus pneumoniae; H. influenzae, Haemophilus influenzae; S. aureus, Staphylococcus aureus; E. coli, Escherichia coli; E. meningospetica, Elizabethkingia meningospetica; M. catarrhalis, Moraxella catarrhalis; E. faecalis, Enterococcus faecalis; S. haemolyticus, Staphylococcus haemolyticus; E. faecium, Enterococcus faecium; A. baumannii, Acinetobacter baumannii; B. multivorans, Burkholderia multivorans; CMV, cytomegalovirus; RSV, Respiratory syncytial virus; HPIV 3, Human parainfluenza 3 virus; HMPV, Human metapneumovirus; HPV 3, Human polyomavirus 3; EBV, Epstein-Barr virus; HRV A, Rhinovirus A; TTV, Torque teno virus; HHV 6B, Human betaherpesvirus 6B; HHV 7, Human betaherpesvirus 7; P. jirovecii, Pneumocystis jirovecii; A. fumigatus, Aspergillus fumigatus; A. flavus, Aspergillus flavus; R. oryzae, Rhizopus oryzae; R. pusillus, Rhizomucor pusillus; A. niger, Aspergillus niger; MP, Mycoplasma pneumonia.

Among all patients, bacteria, viruses, fungi and MP were detected in 17/55 (30.9%), 25/55 (45.5%), 19/55 (34.5%) and 2/55 (3.6%) cases by mNGS, respectively, and the most common species of the detected bacteria, viruses and fungi were *Streptococcus pneumoniae*, CMV and *Pneumocystis jirovecii*, respectively. However, conventional mirobiological methods detected bacteria, viruses, fungi and MP only in 5, 13, 3 and 3 patients. Specifically, culture identified two cases of *Escherichia coli*, one case of *Staphylococcus haemolyticus* and one case of *Acinetobacter baumannii*, which were also reported by mNGS. Besides, one case of *Elizabethkingia meningospetica* was also identified by culture, which was not reported by mNGS. Swear results of BALF reported three cases of fungal infection, which were identified by mNGS as *Rhizomucor pusillus*, *Aspergillus flavus* and *Aspergillus* spp., respectively. Serology tests identified six cases of CMV (5/6 reported by mNGS), three cases of RSV (reported by mNGS), two cases of EBV (reported by mNGS), one case of *human parainfluenza virus* (reported by mNGS) and three cases of MP (2/3 reported by mNGS) (**Figure 1**).

### Mixed Infection

**Figure 2:**
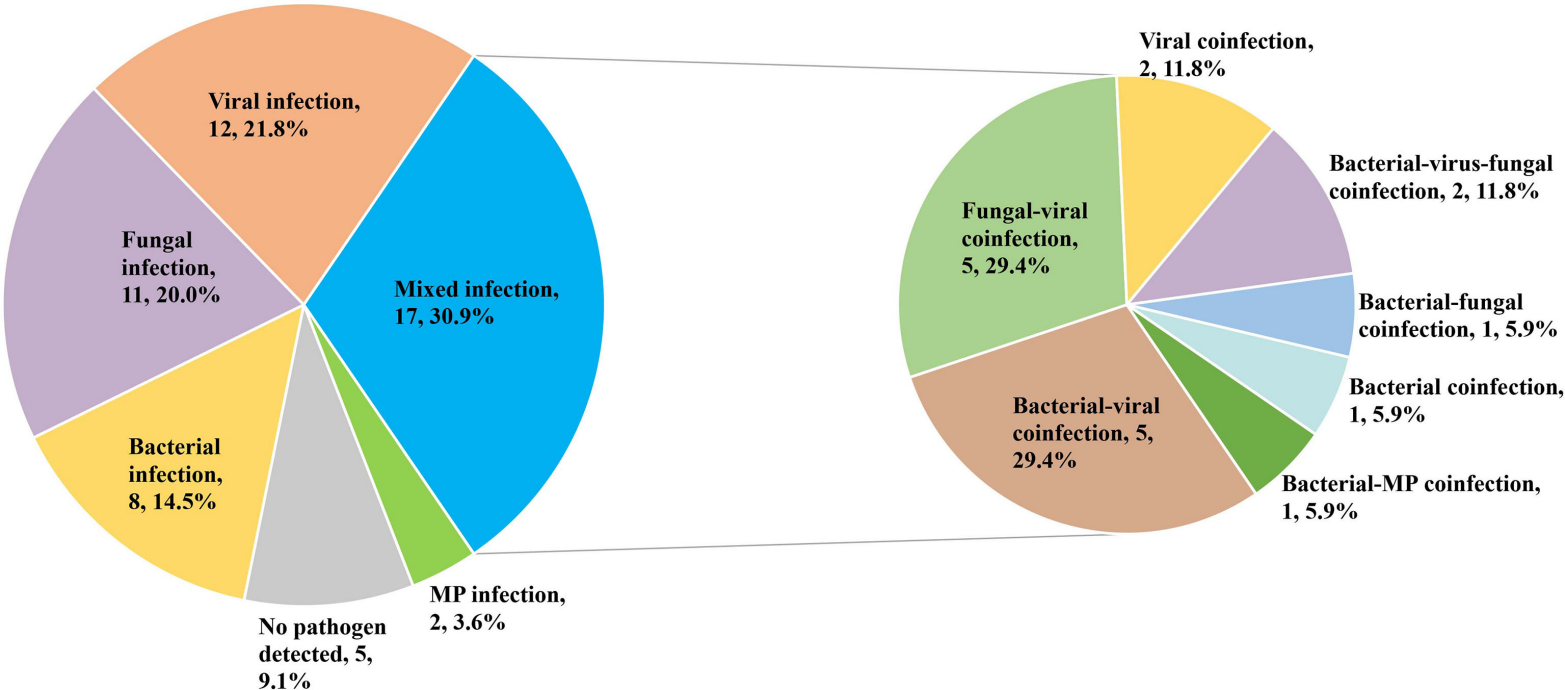
Percentage of patients with mixed infection for various pathogens. MP, Mycoplasma pneumonia.

The mixed infection was defined as more than one pathogenic organism isolated from the same sample. Only 4 patients (7.3%) were identified as a mixed infection using solely conventional pathogen detection methods. When combined with mNGS results, the diagnostic rate of the mixed infection was increased to 30.9% (17/55). The most common mixed infection types were bacterial-viral coinfection (5, 29.4%), fungal -viral coinfection (5, 29.4%) (**Figure 2**).

### The Impact of mNGS on Treatment and Prognosis

**Figure 3:**
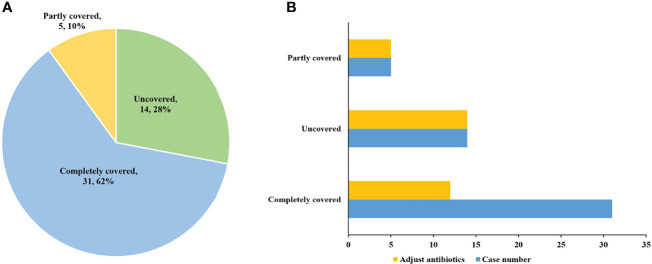
The coverage and adjustment of antibiotics in the patients with pathogen positive pneumonia. **(A)** Among the patients with pathogen positive pneumonia, complete antibiotic coverage was 31 (62%); partial coverage was 5 (10%); and no coverage was 14 (28%). **(B)** Antibiotics were adjusted for 12 completely covered, and for all partly covered and uncovered patients.

Among the 50 patients with pathogen-positive pneumonia, 48 cases were positive for pathogens detected by mNGS. Of the 48 mNGS-positive cases, 29 (60.4%) cases were completely covered by antibiotics before the pathogens were detected. Twelve of these cases had reduced or downgraded antibiotic adjustments after the pathogens were detected, while the other 17 cases did not adjust their antibiotics, of which one case died. The remaining 19 cases were partly covered or not covered by antibiotics before the pathogens were detected, and all these cases adjusted their antibiotics after the pathogens were detected. However, there are 2 cases in uncovered cases died. As for the 2 cases positive for pathogens detected by conventional methods only, empirical antibiotics had completely covered the detected pathogens, and antibiotics were not adjusted after the pathogens were detected, of which one case died (**Figure 3**). Besides, for cases where pathogens were not detected, empirical anti-infection therapy was given and adjusted based on clinical manifestations, imaging findings and other laboratory test results.

## Discussions

Children are in the stage of growth and development, and their immune function is not yet perfect. After suffering from hematologic malignancy, the immune system of their body will be either compromised or destroyed, resulting in immune deficiency. Compared with adults, children are more vulnerable to the invasion of pathogenic microorganisms, thus leading to complex infections. For hematological patients, pulmonary infections are the most common type (Svensson et al., 2017), and the risk of the mixed infection is high, up to 20.5% (Hardak et al., 2016). This is not consistent with our results (31.5%). The reason may be that we did not have a sufficient sample size and our study population is pediatric patients who are more vulnerable to infections. Pulmonary infections can rapidly disseminate, leading to respiratory distress and even death for these children (Erdur et al., 2008; Furuya et al., 2012). In the present study, oxygen therapy was required in 76.4% of pediatric patients and mechanical ventilation was required in 9 patients. Considering the complexity of potential pathogens and the rapid progression of pulmonary infections, rapid pathogenic diagnosis and timely initiation of effective antimicrobial treatment are crucial for better patient survival.

Routine clinical etiological diagnosis can only detect several target pathogens. Due to the diversity of pathogens, routine detection methods have obvious technical limitations in difficult and/or complex infections. mNGS, which does not rely on microbial culture, is based on direct high-throughput sequencing of nucleic acids in clinical samples, and then the sequences are aligned with databases, which include genomic sequences of tens of thousands of species. Determining the species of pathogenic microorganisms (including viruses, bacteria, fungi and parasites) in clinical samples according to the aligned sequence information can be quick and objective. It is especially suitable for the diagnosis of acute and critical diseases and complex infections. Wu et al. showed that the overall microbial yield was 90.3% for BALF mNGS (Wu et al., 2020), which is consistent with our study, where the pathogen-detection yield with BALF mNGS was 87.3%. It indicated that when routine microbiological tests are adverse and empirical treatment is ineffective, BALF mNGS is undoubtedly a valuable pathogen detection tool. Although the utility of bronchoscopy for children remains to be questioned (Rossoff et al., 2019), this procedure positively impacts the pathogenic diagnosis and a subsequent management adjustment of pulmonary infections in this population (Wohlfarth et al., 2018; Bauer et al., 2019). Bronchoalveolar lavage (BAL) is usually more feasible and safer than biopsy (Rao et al., 2013; Chellapandian et al., 2015; Elbahlawan et al., 2016). And mNGS has the potential to improve the diagnostic utility of bronchoscopy (Langelier et al., 2018; Zinter et al., 2019). Taken together, mNGS combined with BAL may be an effective alternative for pathogen detection of pulmonary infections.

Culture and biopsy have been traditionally considered the gold standard for clinical pathogen detection. Still, they are time-consuming, which may delay diagnosis and treatment and consequently contribute to high mortality (Ostrosky-Zeichner, 2012; Khawaja and Chemaly, 2019). In the present study, the workflow from patient sample to results was completed within 24 hours, saving precious time for these critically ill patients. Specifically, the BALF were separately sent to clinical microbiology laboratories within 2 h for analyses, followed by 1 h for nucleic acid extraction from the genome, 15 h for library preparation and sequencing, and 6 h for bioinformatics analyses and reporting. Moreover, mNGS dramatically improved the positive rate of pathogen detection and performed well in mixed infection diagnosis in this study. The overall positivity rate and the rate of mixed infections were 34.5% (19/55) and 7.3% (4/55) only using conventional microbiologic testing. Notably, they increased to 90.9% (50/55) and 30.9% (17/55) when combined with mNGS results, respectively. However, empiric therapy is usually given if pathogens of the infection are unclear, and it sometimes causes ineffective treatment, which brought about increased mortality risk (Ibrahim et al., 2000; Kumar et al., 2006), up to 50% (Wang et al., 2004). Rapid pathogen detection of mNGS can promote timely adjustment of treatment and improve clinical antibiotic overuse in immunocompromised patients. The 50 patients with pathogen-positive pneumonia received timely targeted anti-infection therapy, 12 of whom had reduced or downgraded antibiotic adjustments. Additionally, most of them had better prognosis, and in the present study, the 30-day mortality rate was 7.3%, which indicated that the early diagnosis and prompt treatment through mNGS could significantly decrease the mortality of pediatric patients with HM and pulmonary infections.

Despite its distinct advantage of pathogen detection, mNGS has some drawbacks. The mNGS can theoretically detect all microorganisms in clinical samples without bias simultaneously. However, potential interferences by various colonizing microorganisms from the human body or microorganisms in the external environment in practical applications are challenges to clinical interpretation. For example, bacteria from the oropharyngeal or skin flora (Vanschooneveld et al., 2009), as well as some viruses (such as human herpesviruses (Ho et al., 2020) and Torque teno virus (Wootton et al., 2011)) and most fungi (Romani, 2004), which were not pathogenic in immunocompetent patients, will cause pulmonary infections when the patient’s immune function was low or suppressed. Therefore, the interpretation of pathogens in immunocompromised patients should remain cautious and guided by clinical features and relevant laboratory examination results. Besides, a negative result yielded by mNGS, whilst reducing the chance of an infectious cause, cannot unequivocally exclude infection (Brown et al., 2018). In this study, mNGS failed to identify the causal pathogens for seven cases, in five of which CT imaging and clinical evidence strongly suggested infectious diseases (one case of P. pneumonia, three cases of Gram-positive bacterial and one case of bacterial co-infection), and in two of which infection of *E. meningospetica* and CMV were identified by conventional methods, respectively. For the former five patients, empirical anti-infection therapy was still given and their condition improved. The reason for the false-negative results of mNGS may be that the pathogen load in the specimen is lower than the detection limit of mNGS, leading to false negative detection. Furthermore, pathogens with hard cell walls, such as fungi, may prevent the release of nucleic acid for sequencing, and for the intracellular bacteria, the content of nucleic acids released into extracellular body fluids is low, decreasing the sensitivity of mNGS of both. Thus, even if unique reads of these pathogens yielded by mNGS are not high, the possibility that they are pathogenic should still be considered. As in patient 2, 23 and 51, although the fungi detected by mNGS did not meet the mNGS positive criteria, they were still considered as possible pathogens based on the comprehensive assessment of clinical manifestations and other test results.

There were three limitations in this study. Firstly, due to the retrospective nature of the study, pathogens detected by mNGS were not confirmed by an additional molecular method at a genetic level. Secondly, RNA sequencing was not performed for all samples, which may miss detection of certain pathogens. Thirdly, our study was a small single-center retrospective study, thus there was a selection bias, which still requires more prospective and multicenter data to validate our findings.

In summary, mNGS enables precise and rapid pathogenic diagnosis and makes personalized precision medication feasible, improving clinical outcomes. We propose that mNGS combined with BAL should be performed early in pediatric patients with HM and pulmonary infection. However, the interpretation of mNGS reports should be combined with clinical data and relevant laboratory examination results.

## Data Availability Statement

The data presented in the study are deposited in the CNGBdb, accession number CNP0002773.

## Ethics Statement

The studies involving human participants were reviewed and approved by the First Affiliated Hospital of Zhengzhou University Institutional Review Board (ID: 2021-KY-0405). Written informed consent to participate in this study was provided by the participants’ legal guardian/next of kin.

## Author Contributions

DW conceived and designed the project. LZ, JC, and HY performed the mNGS test. DW, WW, and YD performed bioinformatics analysis. WW and MT collected cases. DW and WW wrote the manuscript. All authors contributed to the article and approved the submitted version.

## Funding

This research was funded by Provincial and Ministerial Co-construction Key Project of Medical Science and Technology of Henan Province (no. SBGJ202002114).

## Conflict of Interest

The authors declare that the research was conducted in the absence of any commercial or financial relationships that could be construed as a potential conflict of interest.

## Publisher’s Note

All claims expressed in this article are solely those of the authors and do not necessarily represent those of their affiliated organizations, or those of the publisher, the editors and the reviewers. Any product that may be evaluated in this article, or claim that may be made by its manufacturer, is not guaranteed or endorsed by the publisher.

## References

[B1] Tomblyn M. Chiller T. Einsele H. Gress R. Sepkowitz K. Storek G.. (2009). Guidelines for Preventing Infectious Complications Among Hematopoietic Cell Transplant Recipients: A Global Perspective. Bone Marrow Transplant. 44, 453–558. doi: 10.1038/bmt.2009.254 19861977

[B2] Bauer P. Chevret S. Yadav H. Mehta S. Pickkers P. Bukan R.. (2019). Diagnosis and Outcome of Acute Respiratory Failure in Immunocompromised Patients After Bronchoscopy. Eur. Respir. J. 54. doi: 10.1183/13993003.02442-2018 31109985

[B3] Brown J. Bharucha T. Breuer J. (2018). Encephalitis Diagnosis Using Metagenomics: Application of Next Generation Sequencing for Undiagnosed Cases. J. infect 76, 225–240. doi: 10.1016/j.jinf.2017.12.014 29305150 PMC7112567

[B4] Chellapandian D. Lehrnbecher T. Phillips B. Fisher B. Zaoutis T. Steinbach W.. (2015). Bronchoalveolar Lavage and Lung Biopsy in Patients With Cancer and Hematopoietic Stem-Cell Transplantation Recipients: A Systematic Review and Meta-Analysis. J. Clin. Oncol. Off. J. Am. Soc. Clin. Oncol. 33, 501–509. doi: 10.1200/jco.2014.58.0480 25559816

[B5] Chen H. Fan C. Gao H. Yin Y. Wang X. Zhang Y.. (2020). Leishmaniasis Diagnosis *via* Metagenomic Next-Generation Sequencing. Front. Cell. infect Microbiol. 10. doi: 10.3389/fcimb.2020.528884 PMC7538539 33072623

[B6] De La Cruz O. Silveira F. (2017). Respiratory Fungal Infections in Solid Organ and Hematopoietic Stem Cell Transplantation. Clinics Chest Med. 38, 727–739. doi: 10.1016/j.ccm.2017.07.013 29128021

[B7] Elbahlawan L. Avent Y. Montoya L. Wilder K. Pei D. Cheng C.. (2016). Safety and Benefits of Bronchoalveolar Lavage and Lung Biopsy in the Management of Pulmonary Infiltrates in Children With Leukemia. J. Pediatr. hematology/oncol 38, 597–601. doi: 10.1097/mph.0000000000000644 PMC5699503 27467366

[B8] Erdur B. Yilmaz S. Oren H. Demircioğlu F. Cakmakç H. Irken G. (2008). Evaluating Pulmonary Complications in Childhood Acute Leukemias. J. Pediatr. hematology/oncol 30, 522–526. doi: 10.1097/MPH.0b013e31816916e4 18797199

[B9] Ewig S. Glasmacher A. Ulrich B. Wilhelm K. Schäfer H. Nachtsheim K. (1998). Pulmonary Infiltrates in Neutropenic Patients With Acute Leukemia During Chemotherapy: Outcome and Prognostic Factors. Chest 114, 444–451. doi: 10.1378/chest.114.2.444 9726728

[B10] Furuya M. Ramírez-Figueroa J. Vargas M. Bernáldez-Ríos R. Vázquez-Rosales J. Rodríguez-Velasco A. (2012). Diagnoses Unveiled by Early Bronchoscopy in Children With Leukemia and Pulmonary Infiltrates. J. Pediatr. hematology/oncol 34, 596–600. doi: 10.1097/MPH.0b013e318240d54b 22322936

[B11] Gu W. Miller S. Chiu C. Y. (2019). Clinical Metagenomic Next-Generation Sequencing for Pathogen Detection. Annu. Rev. Pathol: Mech. Disease 14, 319–338. doi: 10.1146/annurev-pathmechdis-012418-012751 PMC6345613 30355154

[B12] Hardak E. Avivi I. Berkun L. Raz-Pasteur A. Lavi N. Geffen Y.. (2016). Polymicrobial Pulmonary Infection in Patients With Hematological Malignancies: Prevalence, Co-Pathogens, Course and Outcome. Infection 44, 491–497. doi: 10.1007/s15010-016-0873-3 26792011

[B13] Harris B. Morjaria S. Littmann E. Geyer A. Stover D. Barker J.. (2016). Gut Microbiota Predict Pulmonary Infiltrates After Allogeneic Hematopoietic Cell Transplantation. Am. J. Respir. Crit. Care Med. 194, 450–463. doi: 10.1164/rccm.201507-1491OC 26886180 PMC5003327

[B14] Ho D. Y. Enriquez K. Multani A. (2020). Herpesvirus Infections Potentiated by Biologics. Infect. Dis. Clinics 34, 311–339. doi: 10.1016/j.idc.2020.02.006 32444012

[B15] Ibrahim E. Sherman G. Ward S. Fraser V. Kollef M. (2000). The Influence of Inadequate Antimicrobial Treatment of Bloodstream Infections on Patient Outcomes in the ICU Setting. Chest 118, 146–155. doi: 10.1378/chest.118.1.146 10893372

[B16] Khawaja F. Chemaly R. (2019). Respiratory Syncytial Virus in Hematopoietic Cell Transplant Recipients and Patients With Hematologic Malignancies. Haematologica 104, 1322–1331. doi: 10.3324/haematol.2018.215152 31221784 PMC6601091

[B17] Kumar A. Roberts D. Wood K. Light B. Parrillo J. Sharma S.. (2006). Duration of Hypotension Before Initiation of Effective Antimicrobial Therapy is the Critical Determinant of Survival in Human Septic Shock. Crit. Care Med. 34, 1589–1596. doi: 10.1097/01.ccm.0000217961.75225.e9 16625125

[B18] Langelier C. Zinter M. Kalantar K. Yanik G. Christenson S. O'donovan B.. (2018). Metagenomic Sequencing Detects Respiratory Pathogens in Hematopoietic Cellular Transplant Patients. Am. J. Respir. Crit. Care Med. 197, 524–528. doi: 10.1164/rccm.201706-1097LE 28686513 PMC5821905

[B19] Li H. Gao H. Meng H. Wang Q. Li S. Chen H.. (2018). Detection of Pulmonary Infectious Pathogens From Lung Biopsy Tissues by Metagenomic Next-Generation Sequencing. Front. Cell. infect Microbiol. 8. doi: 10.3389/fcimb.2018.00205 PMC6026637 29988504

[B20] Morrison V. (2010). Infectious Complications of Chronic Lymphocytic Leukaemia: Pathogenesis, Spectrum of Infection, Preventive Approaches. Best Pract. Res. Clin. Haematol 23, 145–153. doi: 10.1016/j.beha.2009.12.004 20620978

[B21] Organization W H (2005). Pocket Book of Hospital Care for Children: Guidelines for the Management of Common Illnesses With Limited Resources (World Health Organization). 24006557

[B22] Ostrosky-Zeichner L. (2012). Invasive Mycoses: Diagnostic Challenges. Am. J. Med. 125, S14–S24. doi: 10.1016/j.amjmed.2011.10.008 22196205

[B23] Pendleton K. M. Erb-Downward J. R. Bao Y. Branton W. R. Falkowski N. R. Newton D. W.. (2017). Rapid Pathogen Identification in Bacterial Pneumonia Using Real-Time Metagenomics. Am. J. Respir. Crit. Care Med. 196, 1610–1612. doi: 10.1164/rccm.201703-0537LE 28475350 PMC5754443

[B24] Popovich K. Snitkin E. (2017). Whole Genome Sequencing-Implications for Infection Prevention and Outbreak Investigations. Curr. Infect. Dis. Rep. 19, 15. doi: 10.1007/s11908-017-0570-0 28281083

[B25] Rao U. Piccin A. Malone A. O'hanlon K. Breatnach F. O'meara A.. (2013). Utility of Bronchoalveolar Lavage in the Diagnosis of Pulmonary Infection in Children With Haematological Malignancies. Irish J. Med. Science 182, 177–183. doi: 10.1007/s11845-012-0852-3 22983868

[B26] Renaud C. Campbell A. (2011). Changing Epidemiology of Respiratory Viral Infections in Hematopoietic Cell Transplant Recipients and Solid Organ Transplant Recipients. Curr. Opin. Infect. Diseases 24, 333–343. doi: 10.1097/QCO.0b013e3283480440 21666460 PMC3210111

[B27] Romani L. (2004). Immunity to Fungal Infections. Nat. Rev. Immunol. 4, 11–24. doi: 10.1038/nri2939 14661066

[B28] Rossen J. Friedrich A. Moran-Gilad J. (2018). Practical Issues in Implementing Whole-Genome-Sequencing in Routine Diagnostic Microbiology. Clin. Microbiol. infect Off. Publ. Eur. Soc. Clin. Microbiol. Infect. Diseases 24, 355–360. doi: 10.1016/j.cmi.2017.11.001 29117578

[B29] Rossoff J. Locke M. Helenowski I. Batra S. Katz B. Hijiya N. (2019). Cost Analysis of Bronchoalveolar Lavage and Respiratory Tract Biopsies in the Diagnosis and Management of Suspected Invasive Fungal Infection in Children With Cancer or Who Have Undergone Stem Cell Transplant. Pediatr. Blood Cancer 66, e27598. doi: 10.1002/pbc.27598 30609253

[B30] Salzberg S. Breitwieser F. Kumar A. Hao H. Burger P. Rodriguez F.. (2016). Next-Generation Sequencing in Neuropathologic Diagnosis of Infections of the Nervous System. Neurology(R) Neuroimmunol Neuroinflammation 3, e251. doi: 10.1212/nxi.0000000000000251 PMC4907805 27340685

[B31] Sugawara Y. Nakase K. Nakamura A. Ohishi K. Sugimoto Y. Fujieda A.. (2013). Clinical Utility of a Panfungal Polymerase Chain Reaction Assay for Invasive Fungal Diseases in Patients With Haematologic Disorders. Eur. J. Haematol 90, 331–339. doi: 10.1111/ejh.12078 23360173

[B32] Svensson T. Lundström K. Höglund M. Cherif H. (2017). Utility of Bronchoalveolar Lavage in Diagnosing Respiratory Tract Infections in Patients With Hematological Malignancies: Are Invasive Diagnostics Still Needed? Upsala J. Med. Sci. 122, 56–60. doi: 10.1080/03009734.2016.1237595 27739337 PMC5361433

[B33] Thoendel M. Jeraldo P. Greenwood-Quaintance K. Yao J. Chia N. Hanssen A.. (2018). Identification of Prosthetic Joint Infection Pathogens Using a Shotgun Metagenomics Approach. Clin. Infect. Dis. an Off. Publ. Infect. Dis. Soc. America 67, 1333–1338. doi: 10.1093/cid/ciy303 PMC6186856 29648630

[B34] Vanschooneveld T. Mindru C. Madariaga M. Kalil A. Florescu D. (2009). Enterococcus Pneumonia Complicated With Empyema and Lung Abscess in an HIV-Positive Patient. Case Rep. Rev. literature Int. J. STD AIDS 20, 659–661. doi: 10.1258/ijsa.2008.008456 19710346

[B35] Wang J. Chang Y. Lee L. Chen J. Tang J. Yang P.. (2004). Diffuse Pulmonary Infiltrates After Bone Marrow Transplantation: The Role of Open Lung Biopsy. Ann. Thorac. Surgery 78, 267–272. doi: 10.1016/j.athoracsur.2004.03.002 15223441

[B36] Wohlfarth P. Turki A. Steinmann J. Fiedler M. Steckel N. Beelen D.. (2018). Microbiologic Diagnostic Workup of Acute Respiratory Failure With Pulmonary Infiltrates After Allogeneic Hematopoietic Stem Cell Transplantation: Findings in the Era of Molecular- and Biomarker-Based Assays. Biol. Blood Marrow Transplant. J. Am. Soc. Blood Marrow Transplantat 24, 1707–1714. doi: 10.1016/j.bbmt.2018.03.007 PMC7110883 29550627

[B37] Wootton S. C. Kim D. S. Kondoh Y. Chen E. Lee J. S. Song J. W.. (2011). Viral Infection in Acute Exacerbation of Idiopathic Pulmonary Fibrosis. Am. J. Respir. Crit. Care Med. 183, 1698–1702. doi: 10.1164/rccm.201010-1752OC 21471095 PMC3136996

[B38] Wu X. Li Y. Zhang M. Li M. Zhang R. Lu X.. (2020). Etiology of Severe Community-Acquired Pneumonia in Adults Based on Metagenomic Next-Generation Sequencing: A Prospective Multicenter Study. Infect. Dis. Ther. 9, 1003–1015. doi: 10.1007/s40121-020-00353-y 33170499 PMC7652912

[B39] Zinter M. Dvorak C. Mayday M. Iwanaga K. Ly N. Mcgarry M.. (2019). Pulmonary Metagenomic Sequencing Suggests Missed Infections in Immunocompromised Children. Clin. Infect. Dis. Off. Publ. Infect. Dis. Soc. America 68, 1847–1855. doi: 10.1093/cid/ciy802 PMC6784263 30239621

